# The effect of 34-year continuous fertilization on the SOC physical fractions and its chemical composition in a Vertisol

**DOI:** 10.1038/s41598-019-38952-6

**Published:** 2019-02-21

**Authors:** Zichun Guo, Zhongbin Zhang, Hu Zhou, Daozhong Wang, Xinhua Peng

**Affiliations:** 1 State Key Laboratory of Soil and Sustainable Agriculture, Institute of Soil Science, Chinese Academy of Sciences, Nanjing, 210008 P. R. China; 2 University of Chinese Academy of Sciences, Beijing, 100081 P. R. China; 3 Institute of Soil and Fertilizer Research, Anhui Academy of Agricultural Science, Hefei, 230031 P. R. China

## Abstract

Reports regarding the effects of long-term organic and inorganic fertilization on the quantity and quality of soil organic carbon (SOC), particularly in Vertisols, are scarce. In this study, we combined SOC physical fractionation with ^13^C NMR spectroscopy technology to investigate the effect of 34 years of continuous fertilization on the SOC physical fractions and its chemical composition of 0–20 cm soil layer in a Vertisol. This study consisted of six treatments: no fertilization (control), chemical nitrogen, phosphorus and potassium fertilizers (NPK), low and high amounts of straw with chemical fertilizers (NPKLS and NPKHS), and pig or cattle manure with chemical fertilizers (NPKPM and NPKCM). Over 34 years of continuous fertilization, the SOC sequestration rate was from 0.08 Mg C ha^−1^ yr^−1^ in the control treatment to 0.66 Mg C ha^−1^ yr^−1^ in the NPKCM treatment, which was linearly related with the C input (*P* < 0.01). Of the five SOC physical fractions, two silt plus clay fractions (S + C_M, S + C_mM) dominated 74–92% of SOC, while three POM fractions (cPOM fPOM and iPOM) were only 8–26%. The two manure application treatments significantly increased all the SOC physical fractions except for the silt plus clay fraction within macroaggregates (S + C_M) compared with NPK treatment (*P* < 0.05), which was dependent on the larger amount of C input. Also, the two manure application treatments increased the levels of alkyl C and aromatic C but decreased O-alkyl C (*P* < 0.05), whereas the straw application (NPKLS and NPKHS) had no impact on the C functional groups (*P* > 0.05). Overall, the combination of animal manure with inorganic fertilization could enhance the SOC sequestration and alter its quantity and quality in Vertisols.

## Introduction

The enhancement of SOC sequestration through judicious agricultural management practices is a promising strategy for improving soil fertility and crop yields^1,2^. Fertilization, a common agricultural management practice, can enhance SOC sequestration in cropland soils because it affects the quantity and quality of SOC^3,4^. Although the effects of long-term fertilization on SOC have been extensively reported in many studies, the focus of these works has been predominantly on the total SOC^5–7^ and the effects of fertilization on the quantity and quality of SOC are less defined. Therefore, a better understanding of the effects of long-term fertilization on the chemical composition of SOC and its physical fractions is necessary.

Recently, advanced solid-state ^13^C NMR spectroscopy has come to be universally known as a powerful tool for studying the chemical composition of SOC at a molecular level^8,9^. The chemical composition of SOC is usually divided into four dominant C functional groups: alkyl C, O-alkyl C, aromatic C, and carbonyl C^10^. O-alkyl C is primarily derived from polysaccharides from fresh plant materials that readily decompose^11^. In contrast, both alkyl C and aromatic C mainly consist of original plant biopolymers, soil microbial metabolites and lignin and are regarded as stable organic C^12^. Using this advanced technology, the effects of fertilization on the chemical composition of SOC have been reported in many studies^13–15^. Ultisol amended with pig manure (42 000 kg ha^−1^ yr^−1^) demonstrated more aromatic C, O-alkyl C and carbonyl C than Ultisol amended with straw^15^. Wang *et al*.^15^ also reported obvious differences in the molecular characteristics of SOC, particularly in its density and aggregate fractions, after 4 years of pig-manure compost application in an Anthrosol (paddy soil) in Changshu, China. In contrast, Yan *et al*.^4^ reported that 31 years of continuous application of pig manure (15 000 kg ha^−1^ yr^−1^) had a minimal effect on the chemical composition of SOC in an Anthrosol (paddy soil) in Jinxian, China. These previous reports indicate that, the chemical composition of SOC may be controlled to some extent by the quantity and quality of the organic amendments. Thus, the impact that different quantities and qualities of organic amendments have on the chemical composition of SOC must be assessed via ^13^C NMR spectroscopy technology.

The total SOC is not always a sensitive indicator for detecting changes in or elucidating the mechanisms of SOC sequestration with different agricultural management practices^16^ because SOC is heterogeneous, dynamic and consists of different fractions that vary in their physical and chemical properties, stabilities and turnover rates^17^. To better characterize, predict, and potentially manage SOC sequestration, the total SOC can be generally separated into five fractions by the physical fractionation technique: coarse particulate organic matter (cPOM), fine inter-microaggregate particulate organic matter (fPOM), intra-microaggregate particulate organic matter within macroaggregates (iPOM), silt plus clay fraction within macroaggregate (S + C_M), and silt plus clay fraction within microaggregates occluded within macroaggregates (S + C_mM)^18^. The three POM fractions are a mixture of compounds comprised mainly of plant residues and partial microbial decompositions together, whilst S + C_mM and S + C_M fractions are mineral-associated C (silt and clay protected C). These SOC fractions vary in their sensitivity and responsiveness to the changes induced by fertilization practices. For example, long-term chemical fertilization alone generally had no effect^19–21^ or a positive effect^22^ on SOC fractions compared with unfertilized soil. However, He *et al*.^22^ reported that a combination of straw and chemical fertilization increased the content of cPOM, iPOM, and S + C fractions in an Inceptisol (in Zhengzhou) but decreased in a Mollisol (in Gongzhuling). In contrast, manure application significantly increased all SOC fractions in an Ultisol^23^, Anthrosol^24^ and Inceptisol^22^. Clearly, the effect of various fertilization practices on the SOC physical fractions remains largely unknown.

Vertisols (locally referred to as Shajiang black soil) cover an area of approximately 4 × 10^6^ ha of the Huang-Huai-Hai Plain of China, which is one of the most important wheat production areas in the country. The low SOC content of Vertisols is a major factor for limiting crop yields according to our large-scale survey (data unpublished). To enhance SOC sequestration in Vertisols, the application of straw and manure is encouraged by local policy makers. The long-term application of these amendments plays a critical role in the chemical composition of SOC, the level of each fraction, and subsequently crop yields. We hypothesized that the impact of organic and inorganic fertilization on the SOC sequestration rate and chemical composition would vary, according to differences in the quantity and quality of the amendments used. Therefore, the objectives of this study were to (1) evaluate the long-term effects of fertilization with straw and manure on crop yields and SOC sequestration rates, (2) determine the effect of long-term fertilization on the chemical composition of SOC with ^13^C NMR spectroscopy technology, and (3) determine the response of SOC fractions to different fertilization practices with the SOC physical fractionation technique.

## Materials and Methods

### Site description

The research site is located at the Madian Agro-Ecological station, Anhui Province, in the Huang-Huai Plain of China. This region has a typical sub-humid climate: the average annual temperature is 16.5 °C, and the average annual rainfall is 872 mm. The soil is locally referred to as Shajiang black soil and classified as Vertisol according to the USDA soil taxonomy^25^, and montmorillonite is the dominant clay mineral. This long-term fertilization experiment was initiated in 1982. Prior to the establishment of the experiment (1982), the initial soil in the plough layer (0–20 cm) contained 5.8 g kg^−1^ organic Carbon (C), 0.96 g kg^−1^ total Nitrogen (N), 0.28 g kg^−1^ total Phosphorus (P), 280 g kg^−1^ sand, 306 g kg^−1^ silt, and 414 g kg^−1^ clay, and it had a pH (1:2.5 soil/water) of 7.4 and a bulk density of 1.45 g kg^−1^.

### Experimental design

This long-term fertilization experiment consisted of six treatments with a randomized complete block design. Each treatment had four replicates and each plot was 75 m^2^ (15 m × 5 m). The six treatments were as follows: (1) control, no fertilization, (2) NPK, chemical nitrogen, phosphorus and potassium fertilizers, (3) NPKLS, NPK fertilizers plus 3 750 kg ha^−1^ yr^−1^ wheat straw, (4) NPKHS, NPK fertilizers plus 7 500 kg ha^−1^ yr^−1^ wheat straw, (5) NPKPM, NPK fertilizers plus 15 000 kg ha^−1^ yr^−1^ fresh pig manure, and (6) NPKCM, NPK fertilizers plus 30 000 kg ha^−1^ yr^−1^ fresh cattle manure. The moisture contents of the straw, pig manure and cattle manure were 33.3%, 48% and 58.3%, respectively. The doses of N, P_2_O_5_ and K_2_O applied to the Vertisol were 180 kg ha^−1^, 90 kg ha^−1^ and 135 kg ha^−1^, respectively. All inorganic and organic fertilizers used as basal fertilizers were applied before the sowing of the wheat in October. The crop system was wheat-soybean rotation.

### Crop yield and soil sampling

Wheat or soybean yield was obtained after harvesting the crops of each plot and converted to 14% moisture content for weight calculation. The data of crop yield were from 2012 to 2016. After the soybean harvest in early October, 2016, soil samples were collected from five randomly selected sites of each plot at a depth of 0–20 cm to form a composite sample. Visible pieces of crop debris and roots were removed from the soil sample. The soil samples were air dried, ground to pass through a 2 mm sieve and stored at room temperature for SOC NMR spectroscopy and physical fraction analysis.

### NMR spectroscopy analysis

The chemical composition of SOC was measured with solid-state ^13^C nuclear magnetic resonance (NMR) spectroscopy technology according to the method described in detail by Gonçalves *et al*.^26^. Prior to the NMR analysis, soil samples were pretreated with a 10% hydrofluoric acid (HF) solution to remove paramagnetic components, concentrate their relative C content and increase the spectral quality. Briefly, 5 g of air-dried soil (<2 mm) was transferred into a 100 ml polyethylene tube. After the addition of 40 ml of 10% (w/w) HF, the tubes were closed and then vigorously shaken for 30 s. All tubes were subsequently placed in an incubator shaker for 2 h. After shaking, they were centrifuged at 3000 rpm for 10 min at room temperature. The supernatant was decanted and discarded. The residue was again washed with an equal volume of HF. This procedure was repeated at least 12 times. The remaining soil material was washed four times with distilled water, transferred to a 25 ml polyethylene tube, and then freeze dried. The samples were finally ground to pass through a 0.15 mm plastic sieve for NMR spectroscopy analysis.

**Figure 1:**
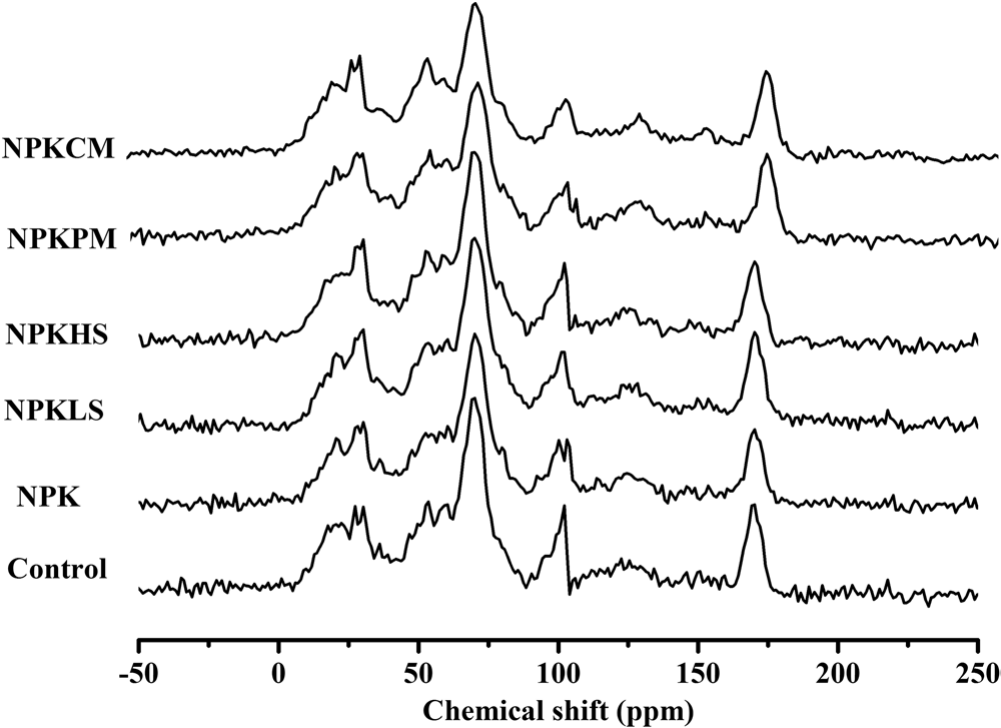
CPMAS-^13^C-NMR spectra of 0–20 cm soil in the Vertisol under different fertilizations.

The CPMAS-^13^C NMR spectroscopy was performed with a Bruker Avance III 400 MHz NMR spectrometer operating at 100.4 MHz. The entire chemical shift region of the ^13^C NMR spectroscopy for each treatment is shown in Fig. 1. According to the chemical shift regions and the spectroscopy assignments described by Kögel-Knabner (1997)^27^, the ^13^C NMR spectral was generally divided into the following C functional groups: (1) alkyl C (0–45 ppm) - terminal methyl groups, methylene groups in aliphatic rings and chains, (2) methoxyl C (45–60 ppm) - methoxyl groups, also classified as O-alkyl C (C-6 for carbohydrates and sugars, C-a for most amino acids), (3) carbohydrate C (60–90 ppm) - carbohydrate-derived structures (C-2 to C-5) in hexoses and higher alcohols (C-a for some amino acids), (4) di-*O*-alkyl C (90–110 ppm) - anomeric carbon of carbohydrates (C-2, C-6 and syringyl units of lignin), (5) aryl C (110–142 ppm) - aromatic C-H carbons (guaiacyl, C-2, C-6 in lignin, and olefinic carbons), (6) phenolic C (142–160 ppm) - aromatic COR or CNR groups, and (7) carboxyl C (160–220 ppm) carboxyl/carbonyl/amide carbons, in which (2), (3) and (4) can be combined as O-alkyl C (45–110 ppm), (5) and (6) as aromatic C (110–160 ppm). The ratio of alkyl C/O-alkyl C was used as an index to assess the degree of SOM decomposition^28^. The aromaticity was used as an index to characterize the extent of humification of the SOM^29^, which was calculated with the following equation:

$${\rm{Aromaticity}}\,( \% )={\rm{Aromatic}}\,{\rm{C}}/({\rm{Aromatic}}\,{\rm{C}}+{\rm{Alkyl}}\,{\rm{C}}+{\rm{O}} \mbox{-} {\rm{alkyl}}\,{\rm{C}})\times 100.$$

### SOC physical fractions

The method used for SOC physical fractionation was adopted from Six *et al*.^12^. The bulk soil was sorted into cPOM, mM, S + C_M by using the wet sieving method. Briefly, 50 g of <2 mm air-dried soil and 50 glass beads (diameter = 4 mm) were placed on a 250 μm sieve. Before wet sieving, each soil sample was soaked in deionized water for 10 min. After removing any floating litter, the sieve was manually agitated 50 times over 2 min (approximately 25 3-cm oscillations min^−1^). After removing the beads form the sieve, any material that was >250 μm (cPOM plus sand) was left on the sieve. All <250 μm materials were then flushed immediately onto a 53 μm sieve with a continuous and steady water flow. The soil remaining on the 53 μm sieve was sieved manually in the same way, to isolate the mM (53–250 μm) and the S + C_M (<53 μm) and transferred in its entirety into an aluminum box.

Next, the 53–250 μm heavy fraction and fPOM were separated by density flotation. Briefly, a 5 g subsample of the microaggregate was oven dried (110 °C) overnight and suspended in 35 ml of 1.85 g cm^−3^ sodium iodide (NaI) in a 100 ml centrifuge tube. The suspension was shaken reciprocally by hand for 30 strokes, and the material remaining on the cap and sides of the centrifuge tube was washed into the suspension twice with 5 ml of sodium iodide. The sample was then put in a vacuum chamber for 10 min. After 20 min of equilibration, the sample was centrifuged at 3500 r min^−1^ for 10 min, and then the supernatant was immediately filtered through a 0.45 μm filter membrane under vacuum and the NaI was collected for reuse. The materials retained on the filter membrane (defined as fPOM) were rinsed into an aluminum box with deionized water at least three times. The heavy fraction remaining in the centrifuge tube was rinsed twice with deionized water, and dispersed in 50 ml of 0.5% sodium hexametaphosphate (HMP) by shaking at 300 r min^−1^ for 18 h on a reciprocal shaker. Finally, the dispersed heavy fraction was passed through a 53 μm sieve to isolate the iPOM (53–250 μm) and S + C_mM (<53 μm). All fractions were dried at 50 °C and weighed. The C contents of various fractions were measured with an elemental analyser (Vario MAX CN, Germany).

### Estimation of the C input and SOC sequestration rate

Th**e** C input (*C*_input_) (Mg ha^−1^ yr^−1^) was estimated according to the stubble and root derived C (*C*_root+stubble_), straw-returned C (*C*_straw_) and manure-applied C (*C*_manure_) with the following equations:

1 $${C}_{{\rm{input}}}={C}_{{\rm{root}}+{\rm{stubble}}}+{C}_{{\rm{straw}}}+{C}_{{\rm{manure}}}$$

2 $${C}_{{\rm{root}}+{\rm{stubble}}}=(({Y}_{{\rm{grain}}}+{Y}_{{\rm{straw}}})\times R\times {R}_{root}+{R}_{stubble}\times {Y}_{straw})\times (1-{\rm{W}})\times O{C}_{{\rm{crop}}}\div1000$$

3 $${C}_{{\rm{straw}}}={B}_{{\rm{straw}}}\times O{C}_{{\rm{straw}}}$$

4 $${C}_{{\rm{manure}}}={B}_{{\rm{maure}}}\times O{C}_{{\rm{manure}}}$$

where *Y*_grain_ and *Y*_straw_ are the grain and straw yields (kg ha^−1^), respectively; 1.1 and 1.6 are the ratios of straw to grain for wheat and soybean, respectively^30^; *R* is the ratio of root biomass to total aboveground biomass (0.429 for wheat and 0.235 for soybean); *R*_root_ is the ratio of the root system within the topsoil (0–20 cm) (0.753 for wheat and 0.984 for soybean); *R*_stubble_ is the coefficient of stubble (0.13 for wheat and 0.15 for soybean); *W* is the water content of air-dried gain (14%); *OC*_crop_ is the C content of air-dried crop (399 g kg^−1^ for wheat and 453 g kg^−1^ for soybean). *B*_straw_ and *B*_maure_ are the straw biomass and manure biomass (kg ha^−1^), respectively; *OC*_straw_, and OC_manure_ are the C contents of wheat straw (482 g kg^−1^), and manure (366 g kg^−1^ for pig manure and 374 g kg^−1^ for cattle manure), respectively^6^. The SOC sequestration rate (Mg ha^−1^ yr^−1^) was calculated with the following equation:

5 $${\rm{SOC}}\,{\rm{sequestration}}\,{\rm{rate}}=({{\rm{SOC}}}_{{\rm{current}}}-{{\rm{SOC}}}_{{\rm{initial}}})\times \rho \times H\times 10/T$$

where SOC_current_ and SOC_initial_ are the content of SOC in 2016 and the initial year (1982), respectively; *ρ* is the soil bulk density (g cm^−3^); *H* is the depth of the soil layer (20 cm); and *T* is the period of the experiment (34 years). The SOC sequestration efficiency (%) was calculated as follows:

6 $${\rm{SOC}}\,{\rm{sequestration}}\,{\rm{efficiency}}\,( \% )={\rm{SOC}}\,{\rm{sequestration}}\,\mathrm{rate}/{\rm{C}}\,{\rm{input}}\times {\rm{100}}$$

where C input is annual C input via stubble and root, straw and manure.

### Statistical analysis

The data analysis was performed with SPSS 22.0 software for Windows (SPSS Inc., USA). The difference in yield, C inputs, and SOC sequestration rate, chemical composition of the SOC and its fractions among the fertilization treatments were assessed with a one-way analysis of variance (ANOVA) and a least significant difference (LSD) test. A simple linear-regression analysis was conducted to reveal the relationships between SOC sequestration and crop yields or C inputs. All analyses were considered significant at *P* < 0.05.

## Results

### Crop yields

**Figure 2:**
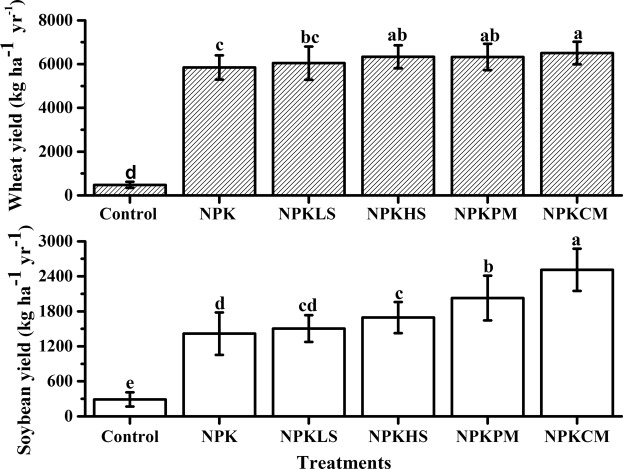
Wheat yield (upper) and Soybean yield (bottom) in the long-term fertilization experiment (2012–2016). Bars on columns are standard deviations (Number = 4). Different letters indicate the difference between fertilization treatments at *P* < 0.05.

The annual wheat and soybean yields with different fertilization treatments from 2012 to 2016 are shown in Fig. 2. The two animal manures combined with NPK fertilization (NPKPM and NPKCM) increased the annual wheat yield by 8.17% and 11.3% relative to the NPK treatment (*P* < 0.05), respectively. The highest increase in the annual soybean yield was also observed in these two treatments, which presented yields of 43.0% and 77.2%, respectively. These values are appreciably larger than those for the NPK treatment (*P* < 0.05). The high amount of straw return (NPKHS) also significantly increased the annual wheat and soybean yields (8.27% and 19.4%, respectively) compared with the effects of NPK fertilization (*P* < 0.05).

### C input and SOC sequestration

**Figure 3:**
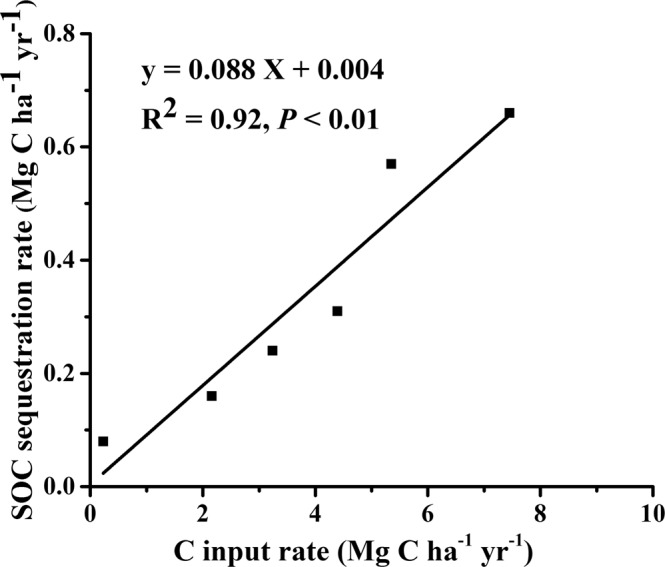
A linear relationship between SOC sequestration rate and C input rate in the Vertisol.

The estimated C inputs and SOC sequestration rates in the plow layer (0–20 cm) for each treatment after 34 years of continuous fertilization are listed in Table 1. The C input in the combined straw or manure treatments with inorganic fertilization (3.24–7.45 Mg C ha^−1^ yr^−1^) was much greater than that in the NPK (2.16 Mg C ha^−1^ yr^−1^) and control (0.23 Mg C ha^−1^ yr^−1^) treatments. The C input in the form of stubble and root for wheat (0.13–1.76 Mg C ha^−1^ yr^−1^) was greater than that of soybean (0.04–0.88 Mg C ha^−1^ yr^−1^) for a given fertilization practice. Consequently, compared to the control treatment, the NPK application increased the SOC content by 1.22 g kg^−1^. Compared with the NPK treatment, the long-term inorganic and organic combined fertilization treatment increased the SOC content by 16–132%, and these values are almost equivalent to the increase in the C storage of 3.02–25.02 Mg C ha^−1^ when relative to the initial SOC content level in 1982 (5.86 g kg^−1^). The long-term inorganic and organic fertilization treatments increased the SOC sequestration rate considerably relative to the unfertilized control treatment (Table 1) (P < 0.05). The smallest increase was observed in the NPK treatment (0.16 Mg C ha^−1^ yr^−1^), while the largest increase was observed in the NPKPM (0.57 Mg C ha^−1^ yr^−1^) and NPKCM (0.66 Mg C ha^−1^ yr^−1^) treatments. A linear relationship between the C input and SOC sequestration rate is shown in Fig. 3 (R^2^ = 0.92, *P* < 0.01). The highest SOC sequestration efficiency was observed in the control treatment (33.4%). The SOC sequestration efficiencies for the inorganic and organic fertilization (NPKLS, NPKHS, NPKPM and NPKCM) varied from 7.12% to 10.7%, but a significant difference was not observed among the fertilization treatments (*P* > 0.05) (Table 1).

**Table 1:** C inputs and SOC sequestration in the 0–20 cm soil layer of the Vertisol under different fertilization treatments (Mean ± SD, n = 4).

Treatments	Stubbles and roots	Organic Amendments	Total C inputs	SOC^δ^	Bulk density	SOC sequestration rate	SOC Sequestration efficiency
	(Mg C ha^−1^ yr^−1^)			(g kg^−1^)	(g cm^−3^)	(Mg C ha^−1^ yr^−1^)	%
Control	0.23 ± 0.01 e	0	0.23 ± 0.01 f	7.52 ± 0.23 f	1.31 ± 0.03 ab	0.08 ± 0.01 f	33.4 ± 5.96 a
NPK	2.16 ± 0.08 d	0	2.16 ± 0.08 e	8.70 ± 0.13 e	1.28 ± 0.01 ab	0.16 ± 0.01 e	7.28 ± 0.76 b
NPKLS	2.24 ± 0.07 d	1.00	3.24 ± 0.07 d	10.1 ± 0.20 d	1.24 ± 0.08 b	0.24 ± 0.05 d	7.37 ± 1.56 b
NPKHS	2.39 ± 0.06 c	2.00	4.39 ± 0.06 c	11.4 ± 0.16 c	1.21 ± 0.06 bc	0.31 ± 0.05 c	7.12 ± 1.21 b
NPKPM	2.49 ± 0.01 b	2.86	5.35 ± 0.01 b	14.7 ± 0.01 b	1.24 ± 0.04 b	0.57 ± 0.03 b	10.7 ± 0.59 b
NPKCM	2.70 ± 0.08 a	4.75	7.45 ± 0.08 a	17.5 ± 0.32 a	1.13 ± 0.04 c	0.66 ± 0.05 a	8.90 ± 0.60 b

^δ^SOC: soil organic carbon.

### SOC chemical composition

The C functional groups under fertilization, are shown in Table 2. The ^13^C NMR spectroscopy results showed that O-alkyl C (50.9–55.2%) predominated, followed by alkyl C (22.5–26.1%), aromatic C (12.2–14.3%) and carbonyl C (8.6–10.0%). An increase in the proportion of alkyl C and a decrease in that of O-alkyl C in the NPKPM and NPKCM treatments were observed compared with the levels in the NPK treatment (*P* < 0.05). The ratio of alkyl C/O-alkyl C and the aromaticity were all greater in the NPKPM and NPKCM treatments than in the other fertilization treatments.

**Table 2:** Relative intensities of various C functional groups in CPMAS-^13^C-NMR spectra in the 0–20 cm soil layer of the Vertisol under different fertilization treatments (Mean ± SD, n = 4).

Treatments	Alkyl C	O-alkyl C	Aromatic C	Carbonyl C	Alkyl C/O-alkyl C	Aromaticity (%)
	(0–45)	(45–110)	(110–160)	(160–220)		
	(%)					
Control	24.4 ± 0.96 b	54.6 ± 1.21 a	12.3 ± 1.45 b	8.60 ± 0.50 a	0.45 ± 0.01 b	14.1 ± 1.65 b
NPK	23.7 ± 0.46 b	54.3 ± 0.95 a	12.4 ± 0.98 b	9.67 ± 0.46 a	0.44 ± 0.00 b	14.1 ± 1.15 b
NPKLS	22.5 ± 1.79 b	55.2 ± 1.69 a	12.4 ± 1.56 b	10.0 ± 1.42 a	0.41 ± 0.03 b	14.2 ± 1.87 b
NPKHS	23.2 ± 1.96 b	55.2 ± 0.70 a	12.2 ± 0.75 b	9.37 ± 1.04 a	0.42 ± 0.04 b	13.9 ± 0.98 b
NPKPM	25.5 ± 1.32 a	51.6 ± 0.75 b	13.7 ± 0.40 a	9.27 ± 1.64 a	0.49 ± 0.02 a	15.9 ± 0.73 a
NPKCM	26.1 ± 0.17 a	50.9 ± 0.60 b	14.3 ± 0.46 a	8.80 ± 0.26 a	0.51 ± 0.01 a	16.6 ± 0.49 a

### SOC physical fractions

The SOC fractions were greatly altered by long-term fertilization (Table 3). The total SOC recovery from the bulk soil after wet sieving, density flotation and dispersion was 90% to 98%. Of the five SOC fractions, the S + C_mM fraction predominated at 42–50%, followed by the S + C_M fraction (20–42%) and then the iPOM fraction (4–12%), fPOM fraction (2–10%) and cPOM fraction (2–4%). The inorganic fertilization treatment (NPK) significantly increased the content of all SOC fractions except for the cPOM fraction compared with the unfertilized treatment (control) (*P* < 0.05). The combined organic and inorganic fertilization treatment (NPKHS, NPKPM and NPKCM) notably increased the content of the cPOM (57–238%), fPOM (77–313%), iPOM (74–319%) and S + C_mM (32–130%) fractions (*P* < 0.05) relative to that of the NPK treatment but did not increase the content of the S + C_M fraction (*P* > 0.05). Carbon inputs were linearly related to the cPOM (R^2^ = 0.88, *P* < 0.01), fPOM (R^2^ = 0.94, *P* < 0.01), iPOM (R^2^ = 0.89, *P* < 0.01) and S + C_mM (R^2^ = 0.89, *P* < 0.01).

**Table 3:** SOC physical fractions associated C of the 0–20 cm soil layer in the Vertisol under different fertilization treatments (Mean ± SD, n = 4).

Treatments	cPOM^α^	Microaggregates (53–250 μm)			S + C_M^ε^
		fPOM^β^	iPOM^γ^	S + C _mM^δ^	
	g kg^−1^				
Control	0.12 ± 0.03 d	0.17 ± 0.05 e	0.30 ± 0.02 f	3.37 ± 0.16 f	3.05 ± 0.12 b
NPK	0.19 ± 0.04 cd	0.40 ± 0.04 d	0.48 ± 0.05 e	3.81 ± 0.09 e	3.66 ± 0.23 a
NPKLS	0.18 ± 0.03 d	0.62 ± 0.22 cd	0.61 ± 0.07 d	4.23 ± 0.14 d	3.68 ± 0.13 a
NPKHS	0.28 ± 0.06 c	0.71 ± 0.08 c	0.84 ± 0.06 c	5.03 ± 0.11 c	3.67 ± 0.21 a
NPKPM	0.47 ± 0.13 b	1.01 ± 0.17 b	1.14 ± 0.05 b	7.15 ± 0.47 b	3.43 ± 0.19 a
NPKCM	0.61 ± 0.04 a	1.65 ± 0.15 a	2.01 ± 0.15 a	8.78 ± 0.29 a	3.47 ± 0.17 a

^α^cPOM: coarse particulate organic matter; ^β^fPOM: fine inter-microaggregate POM; iPOM: intra-microaggregate POM within macroaggregates;

^δ^S + C_mM: silt plus clay fraction within microaggregates occluded within macroaggregates; ^ε^S + C_M: silt plus clay within macroaggregates.

## Discussion

### Crop yield in the Vertisol under long-term fertilization

The annual yield of both wheat and soybean increased after long-term fertilization compared with no fertilization (*P* < 0.05). In particular, the greatest increase in yield for both wheat and soybean was observed in the animal manure application treatments (NPKPM and NPKCM). Similar findings have been observed in previous studies^31,32^. The positive effects of manure application on crop yield are likely due to the improved physical environment of the soil coupled with the increased availability of the nutrients necessary for crop growth^33,34^. Increases in crop yield with a simultaneous increase in SOC have been reported in several cropping systems by numerous researchers^35,36^. In this study, regression analysis showed that the mean increases in wheat (R^2^ = 0.77, *P* < 0.05) and soybean (R^2^ = 0.94, *P* < 0.01) yields were linearly correlated with increased SOC sequestration, which indicated that the SOC is one of the most important factors of crop production in a Vertisol. Although the soybean crop did not receive any fertilization after the wheat harvest, the soybean yield showed a more obvious increase than the wheat yield did, compared to the effects of inorganic fertilizer alone treatments following 34 continuous years of fertilization in this study. This finding suggests that obtaining high yields in a rainfed wheat-soybean system depends not only on nutrients but also on other factors including plant water availability. In fact, approximately two-thirds of the annual rain fall occurs from June to September during the soybean growing season, while only one-third falls from October to May, during the winter wheat growing season^37^. Thus, both appropriate fertilization practices and better environment conditions need to be seriously addressed for the development of sustainable agriculture in a Vertisol.

### Relationship between the C input and SOC sequestration in the Vertisol

The amount of C sequestered in the Vertisol increased considerably (16–132%) after 34 years of continuous organic fertilization compared with inorganic fertilization, which is consistent with several previous studies^4,20,21^. Long-term fertilization increased the SOC sequestration in at least two distinct ways. Firstly, straw and manure themselves act as an exogenous C source contributing to SOC sequestration, and secondly, increasingly more balanced fertilization may result in better plant growth, which may in turn result in a more pronounced rooting system. Increased plant growth due to balanced plant nutrition may also result in higher amounts of crop residues that are returned to the soil after harvest. Interestingly, the unfertilized control treatment also increased SOC content by 28% when compared to the initial condition. This finding is consistent with the results reported by Bhattacharyya *et al*.^38^, who showed that the cultivation of wheat and soybean for 30 years in an Inceptisol in the Indian Himalayas without any added organic and/or inorganic fertilizers increased the SOC content by 9%. The C from wheat crop residues in the unfertilized control treatment was estimated at 140 kg ka^−1^ yr^−1^ and that from soybean residues was estimated at 90 kg ha^−1^ yr^−1^, which may be enough to mitigate C losses via SOM decomposition^39^.

In most cases, the relationship between C inputs and C sequestration is best fit with a logarithmic equation, particularly when the C input range is wide, while a linear relationship can occur with a narrow range of C inputs^40^. Our result (0.16–7.39 Mg C ha^−1^ yr^−1^) showed a linear relationship (Fig. 3), as did the results reported by Zhang *et al*.^41^ across six sites in China (0.81–11.1 Mg C ha^−1^ yr^−1^). This linear relationship implies a continuous increase of SOC stock with increasing C inputs^40,42^. Thus, Vertisols still have great potential for C sequestration. Compared with the NPK treatment, the combined animal manure and inorganic fertilization treatments increased the SOC sequestration by 19.6–43.8%. Our results are consistent with a number of studies based on long-term fertilization experiments^20,21^ but inconsistent with others^23,43^ in which the SOC sequestration efficiency under combined organic and inorganic fertilization treatments was almost half that in the inorganic fertilization alone treatments. This is because the mechanisms controlling SOC sequestration efficiency are complex and are affected not only by soil properties^44^ but also by the quantity and quality of the C input, C stabilization and the saturation deficit^6,45^.

### Chemical composition of SOC in the Vertisol

Differences in the proportions of C functional groups between unfertilized and fertilized soils may be strongly related to the amount of C inputs^46^. However, our results suggest that the quality of C input might directly influence the C functional group proportions. Compared with the control or NPK treatment, we found that the animal manure application treatments (NPKPM and NPKCM) increased alkyl C and aromatic C but decreased *O*-alkyl C, while the straw application treatments (NPKLS and NPKHS) had no impact on the C functional groups. Both animal manures were composted before application so that any readily decomposed C was lost, which resulted in increase in recalcitrant C (i.e., alkyl C, aromatic C) and decrease in easily decomposed C (i.e., O-alkyl C)^47–49^. Similar results have been reported in an Anthrosol by Zhou *et al*.^50^, who observed the negative correlation of recalcitrant C with easily decomposed C after the long-term application of animal manures. These results indicate that after animal manure application, recalcitrant C is preferentially preserved during the process of SOC sequestration relative to other C functional groups, which leads to improved SOC stability. Thus, the enhancement of SOC sequestration though animal manure application to a Vertisol shifts the accumulation of C in favour of recalcitrant C. In addition, we also found that the ratio of alkyl C/O-alkyl C was higher in the animal manure application treatments than the other fertilization treatments, which is consistent with the study by Wang *et al*.^15^ in a rice-wheat cropping system. The higher ratio of alkyl C/O-alkyl C after animal manure application suggests that this treatment may accelerate the decomposition of the O-alkyl C contained in manures into soil, which results in soil being unable to effectively accumulate labile organic C^51^. Composting animal manure before application is therefore a necessary part of cropland management^52^. From the perspective of C sequestration, reducing the loss of easily decomposed C during the composting process is desirable.

### Sequestration mechanisms of SOC fractions in the Vertisol

SOC sequestration is jointly controlled by three principal mechanisms: (i) the molecular recalcitrance of OM, (ii) the physical protection of SOC, and (iii) the biochemical protection of SOC^53^. The proportions of the cPOM and fPOM fractions varied from 4% to 14% of the total SOC in this study, which correspond reasonably well with Christensen^54^, who stated that these two fractions generally accounted for less than 10% of the total SOC in the plough layer in cropland soils. The highest increase in the content of cPOM and fPOM fractions was observed in the NPKPM (161% and 238%, respectively) and NPKCM (152% and 313%, respectively) treatments (Table 3). This may be due to the recalcitrance of the manure under higher amounts of C input^55^. The decomposed manure contained more recalcitrant C at a molecular level as mentioned above. In addition, long-term manure application considerably increased the content of the iPOM (138% and 319%, respectively) and S + C_mM (88% and 130%, respectively) fractions via physical and biochemical protection, thereby further increasing the long-term C sequestration in a Vertisol. Our results are consistent with some previous works^20,56^, which is likely due to the ability of manure application to promote the formation of microaggregates within macroaggregates^57,58^.

**Figure 4:**
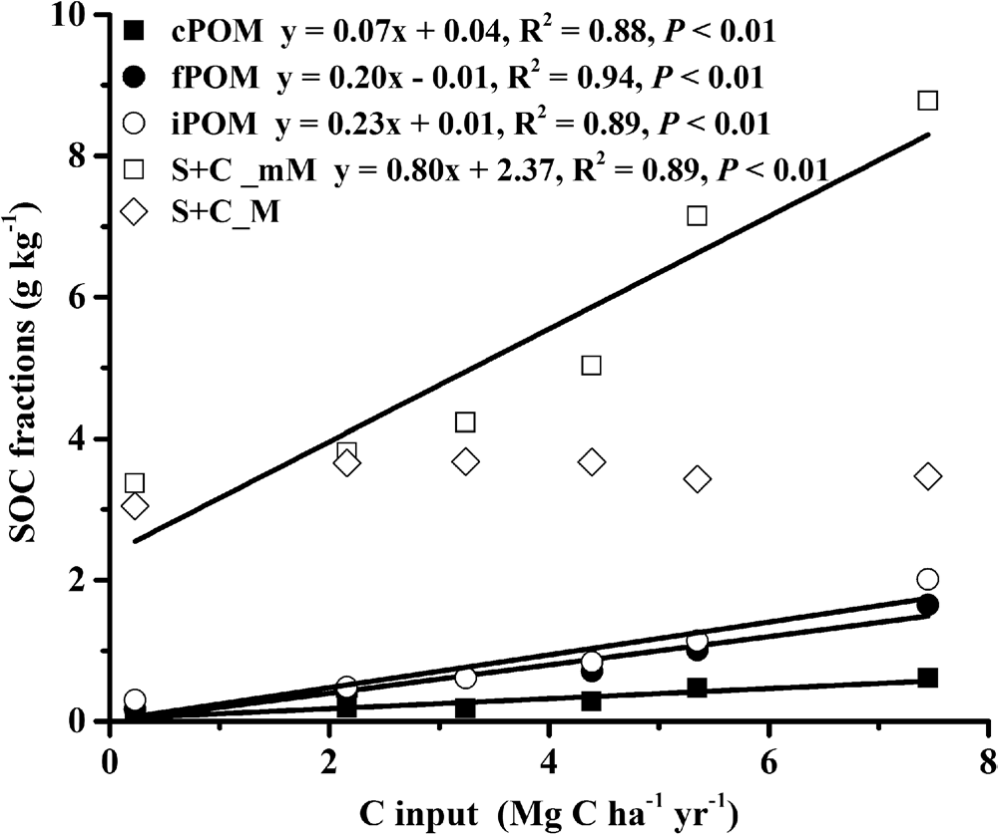
Relationship between SOC fractions and C input rate in the Vertisol.

Carbon inputs are the dominant driver for sequestering C in soil^39^. Four SOC fractions (cPOM, fPOM iPOM and S + C_mM) did exhibited a linear relationship with the C inputs in this study (Fig. 4), which indicates that these SOC fractions still have a substantial C saturation deficit, and could still stabilize the additional amounts of C inputs, thereby continuing to act as atmospheric C sinks^59,60^. Among these four fractions, the S + C_mM fraction was the most sensitive to fertilization practices and hence could be used as a diagnostic fraction for future management-induced changes of SOC in Vertisols. Furthermore, the differences in the responsivity of each SOC fractions also indicates that shifting the SOC towards relatively more labile organic C by increasing C inputs to Vertisols is a viable long-term fertilization management practice.

## Conclusions

Based on thirty-four years of the continuous application either of straw or manure in a Vertisol, the straw return management enhanced the SOC sequestration but did not alter the SOC quality relative to the unfertilized control treatment. However, application of pig or cattle manure increased SOC storage as well as the contents of alkyl C and aromatic C due to the high quality of the C input. Relative to the straw amendments, manure application significantly increased the SOC physical fractions, particularly in the POM and aggregate associated with the silt and clay C fraction through greater C input. As a result, the SOC sequestration efficiency in the straw amendment treatments was the same as that in the inorganic fertilization alone treatment but lower than that in the manure amendment treatments. Our results indicated that a balanced application of NPK fertilizers with manure should be encouraged to improve SOC sequestration and ensure greater agricultural production in Vertisols.
